# Bye-Laws of the Royal College of Surgeons in London

**Published:** 1831

**Authors:** 


### LXIX.

### BYE-LAWS OF THE ROYAL COLLEGE OF SURGEONS IN LONDON.

No business whatever shall be transacted nor any matter be discussed or debated at any meeting or assemblage convened by or under the authority of the President or Council, or before or after the business thereof shall have commenced, other than the particular business or matter in respect of which such meeting or assemblage shall have been convened; nor shall any debate or discussion whatsoever be had or allowed at any meeting convened by the President or Council for the delivery of Lectures or Orations either before or after the same shall have commenced or terminated. And no meeting or assemblage of Members of the College shall be held in the Hall or Council House of the College, or in any of its appurtenances, unless convened by or under the authority of the President or Council: and no Member of the College shall advertise or convene or attend, or combine with others to advertise or convene or attend any meeting or assemblage in the Hall or Council House of the College, or in any of its appurtenances, not authorized by the President or Council. And any Member of the College who may in any manner offend herein shall be liable to be restrained and excluded by the Council from attending any Orations and Lectures at the Theatre, and from any use of or admission to the Library and Museum, and to be suspended from any or all other Privileges which he may have as a Member of the College, for any such period as the Council may adjudge, or to removal by the Council from being a Member of the College. And every Member of the College who shall thereupon be removed as aforesaid shall forfeit all his rights and privileges as a Member thereof.

All meetings convened by or under the authority of the President or Council of the College, as well for general business as for the delivery of Orations or Lectures, or for the distribution of Prizes, shall be under the controul and direction of the President or other Member of the Council presiding at such Meeting. And any Member of the College who shall interrupt, impede or interfere with the proceedings at any such Meeting, or shall propose any matter for discussion or debate without the leave of the President or other Person so presiding, shall, upon being required by the President or other Person so presiding, immediately withdraw from such Meeting; and shall be moreover liable to be restrained and excluded by the Council from attending any Orations and Lectures at the Theatre, and from any use of or admission to the Library and Museum, and to be suspended from any or all other privileges which he may have as a Member of the College, for any such period as the Council may adjudge. And any Member of the College who shall so offend a second time, or during any suspension by the Council shall attempt to exercise any of the privileges from which he shall be suspended, shall be liable to removal, by the Council from being a Member of the College. And every Member of the College who shall thereupon be removed as aforesaid, shall forfeit all his rights and privileges as a Member thereof.

---

Made and Ordained Bye-Laws of the ROYAL COLLEGE OF SURGEONS IN LONDON by and at a Meeting of the Council of the said ROYAL COLLEGE, holden, at the College, on the 27th day of April, 1831.

---

*We have examined and do approve of and allow these Bye-Laws:—*
BROUGHAM, C.
TENTERDEN,
N. C. TINDAL.

*21st day of May*, 1831.

